# The Insulin-Sensitizer Pioglitazone Remodels Adipose Tissue Phospholipids in Humans

**DOI:** 10.3389/fphys.2021.784391

**Published:** 2021-12-02

**Authors:** Juan P. Palavicini, Alberto Chavez-Velazquez, Marcel Fourcaudot, Devjit Tripathy, Meixia Pan, Luke Norton, Ralph A. DeFronzo, Christopher E. Shannon

**Affiliations:** ^1^Division of Diabetes, Department of Medicine, University of Texas Health Science Center at San Antonio, San Antonio, TX, United States; ^2^Barshop Institute for Longevity and Aging Studies, University of Texas Health Science Center at San Antonio, San Antonio, TX, United States

**Keywords:** adipose, obesity, lipidomics, pioglitazone, type 2 diabetes

## Abstract

The insulin-sensitizer pioglitazone exerts its cardiometabolic benefits in type 2 diabetes (T2D) through a redistribution of body fat, from ectopic and visceral areas to subcutaneous adipose depots. Whereas excessive weight gain and lipid storage in obesity promotes insulin resistance and chronic inflammation, the expansion of subcutaneous adipose by pioglitazone is associated with a reversal of these immunometabolic deficits. The precise events driving this beneficial remodeling of adipose tissue with pioglitazone remain unclear, and whether insulin-sensitizers alter the lipidomic composition of human adipose has not previously been investigated. Using shotgun lipidomics, we explored the molecular lipid responses in subcutaneous adipose tissue following 6months of pioglitazone treatment (45mg/day) in obese humans with T2D. Despite an expected increase in body weight following pioglitazone treatment, no robust effects were observed on the composition of storage lipids (i.e., triglycerides) or the content of lipotoxic lipid species (e.g., ceramides and diacylglycerides) in adipose tissue. Instead, pioglitazone caused a selective remodeling of the glycerophospholipid pool, characterized by a decrease in lipids enriched for arachidonic acid, such as plasmanylethanolamines and phosphatidylinositols. This contributed to a greater overall saturation and shortened chain length of fatty acyl groups within cell membrane lipids, changes that are consistent with the purported induction of adipogenesis by pioglitazone. The mechanism through which pioglitazone lowered adipose tissue arachidonic acid, a major modulator of inflammatory pathways, did not involve alterations in phospholipase gene expression but was associated with a reduction in its precursor linoleic acid, an effect that was also observed in skeletal muscle samples from the same subjects. These findings offer important insights into the biological mechanisms through which pioglitazone protects the immunometabolic health of adipocytes in the face of increased lipid storage.

## Introduction

Adipose tissue is the primary site for fat storage and quantitatively the most important energy reservoir in the body. The coordinated expansion and breakdown of adipose lipid stores is crucial to the dynamic regulation of circulating nutrient availability and plays a central role in the control of whole-body metabolism. Excessive nutrient storage in adipose tissue (i.e., obesity) promotes adipocyte dysfunction, inflammation, and insulin resistance and is therefore strongly implicated in the etiology of type 2 diabetes (T2D; DeFronzo, 2004).

Pioglitazone is an insulin-sensitizing drug approved for the treatment of T2D. The molecular effects of pioglitazone are primarily mediated through the nuclear receptor peroxisome proliferator-activated receptor gamma (PPARγ), a transcriptional regulator of adipocyte differentiation and lipid storage which is highly abundant in adipose tissue (Spiegelman, 1998). Although its precise therapeutic mechanism remains controversial, the disease-modifying actions of pioglitazone are traditionally ascribed to the PPARγ-mediated expansion of subcutaneous adipose tissue, resulting in a reduction in systemic lipid concentrations and the subsequent reversal of “lipotoxicity” in non-adipose tissues (Bays et al., 2004). In the absence of a negative energy balance (i.e., weight loss), the mobilization of ectopic lipids, especially from skeletal muscle (Bajaj et al., 2010) and liver (Bajaj et al., 2003), as well as the redistribution of visceral fat, is accompanied by a reciprocal increase in the subcutaneous adipose tissue depots (Miyazaki et al., 2002) and in adiponectin levels (Gastaldelli et al., 2021). Mechanistically, recent estimates of adipogenesis in obese individuals treated with pioglitazone (White et al., 2021) confirm earlier morphological observations (McLaughlin et al., 2010) that thiazolidinediones drive adipose expansion by stimulating the formation of new adipocytes (i.e., hyperplasia). As a result of this mode of action, pioglitazone paradoxically causes weight gain (specifically fat mass) despite improving dyslipidemia, insulin sensitivity, and glycemic control (Miyazaki et al., 2001; Shadid and Jensen, 2003).

Recent reports have suggested that the expansion of adipose tissue following pioglitazone treatment could be driven by the formation of new adipocytes (i.e., adipogenesis) in subcutaneous adipose depots (White et al., 2021). This is consistent with observations that pioglitazone increases the proportion of smaller adipocytes in subcutaneous adipose tissue (de Souza et al., 2001; McLaughlin et al., 2010) which likely contributes to the enhancement of adipocyte glucose uptake and greater overall capacity for lipid storage (Olefsky, 1976). Importantly, whereas adipose tissue expansion in obesity is coupled with metabolic dysfunction and chronic, low-grade inflammation (Zatterale et al., 2019), the pioglitazone-mediated increase in lipid storage is associated with the promotion of anti-inflammatory pathways in human adipose (Koppaka et al., 2013; Spencer et al., 2014).

The lipid composition of human adipose tissue is dominated by triglyceride species (Al-Sari et al., 2020), but also includes numerous less abundant lipid molecules that may nevertheless be important effectors of inflammatory and insulin signaling pathways. For example, it was recently found that changes in adipose glycerophospholipids, rather than triglycerides, more closely reflect the transcriptional and metabolic adaptations occurring during adipose expansion with diet-induced obesity (Liu et al., 2020). Alterations in certain glycerophospholipid species, including those enriched in arachidonic acid, have been directly implicated in the inflammatory milieu of adipose tissue in human obesity (Pietiläinen et al., 2011). How the molecular lipid profile of adipose tissue responds to pioglitazone therapy has not previously been investigated and, as such, the events involved in adipose tissue remodeling following pioglitazone treatment remain poorly characterized.

The objective of the present study was to determine the impact of pioglitazone treatment on molecular lipids in adipose tissue from obese type 2 diabetics, using a multi-dimensional mass spectrometry-based shotgun lipidomics approach (Han and Gross, 2005), which facilitated the class-targeted analysis of all glycerophospholipid, sphingolipid, acylcarnitine, free fatty acid, triacylglycerol, and diacylglycerol species. Understanding how adipose tissue biology influences the resolution of human insulin resistance can identify novel pathophysiological lipid species and represents an important step toward developing more effective therapeutic strategies to combat the clinical and socioeconomic burden of soaring rates of obesity and T2D.

## Materials and Methods

### Human Studies

Seven obese individuals with T2D (male/female 6/1; Mexican American/Caucasian 5/2; age 57±7years; BMI 32±6kg/m^2^; HbA_1c_ 8.0±0.6%) treated with diet alone or diet plus metformin and/or sulfonylurea participated in the study, which was approved by the Institutional Review Board of the South Texas Veterans Healthcare System, University of Texas Health Science Center San Antonio. After providing fully informed consent and completing a routine health screening visit, eligible subjects reported to The Bartter Clinical Research Unit of the South Texas Veterans Healthcare System following an overnight ~10-h fast. A baseline blood sample was drawn for the measurement of fasting blood glucose, HbA1c, and triglycerides, and subcutaneous abdominal adipose tissue and vastus lateralis muscle biopsies were obtained under local anesthesia (1% Lidocaine) for lipid profiling and gene expression analysis. Baseline measurements were repeated after 6months of pioglitazone treatment (45mg/day). Due to biopsy sample availability, lipidomic analyses were carried out on six of the seven subjects for adipose tissue and five of the seven subjects for skeletal muscle tissue, such that lipidomic analyses in at least one tissue are presented for all subjects. Clinical and adipose gene expression data are presented for all seven subjects.

### Multi-Dimensional Mass Spectrometry-Based Shotgun Lipidomics

Adipose tissue or skeletal muscle samples (10–20mg) were homogenized in ice-cold diluted (10%) phosphate-buffered saline, and lipids were extracted by a modified Bligh and Dyer procedure in the presence of internal standards added based on total protein content, as previously described (Han and Gross, 2005; Wang et al., 2017; Palavicini et al., 2020). A triple-quadrupole mass spectrometer (Thermo Scientific TSQ Altis, CA, United States) and a Quadrupole-Orbitrap^™^ mass spectrometer (Thermo Q Exactive^™^) equipped with a Nanomate device (Advion Biosciences Ltd., NY, United States) and Xcalibur system software were used as previously described (Wang et al., 2017). Briefly, diluted lipid extracts were directly infused into the electrospray ionization source through a Nanomate device. Signals were averaged over a 1-min period in the profile mode for each full-scan mass spectrometry (MS) spectrum. For tandem MS, a collision gas pressure was set at 1.0 mTorr, but the collision energy varied with the classes of lipids. Similarly, a 2- to 5-min period of signal averaging in the profile mode was employed for each tandem MS mass spectrum. All full and tandem MS mass spectra were automatically acquired using a customized sequence subroutine operated under Xcalibur software. Data processing, including ion peak selection, baseline correction, data transfer, peak intensity comparison, 13C deisotoping, and quantitation, was conducted using a custom programmed Microsoft Excel macro as previously described after considering the principles of lipidomics (Yang et al., 2009).

### Adipose Tissue Gene Expression

Target mRNA expression was determined in adipose tissue lysates by qRT-PCR as previously described (Shannon et al., 2017) using the following pre-designed SYBR green human primer assays from Sigma (MO, United States): *FADS1*, *FADS2*, *ELOVL5*, *CDS1*, *CDIPT*, *PLA2G4A*, *PLA2G4C*, *PLA2G7*, and *PLA2G16*. Data were normalized to the geometric mean of the reference genes *ACTB* and *GAPDH* and expressed as a fold change relative to the baseline (pre-treatment) mean.

### Statistical Analyses

Clinical data and lipid class totals are expressed as mean±standard error and were compared using paired *t* tests. Overall lipidomics data patterns were initially visualized by principal component analyses (PCA) performed on raw data for quantified species (in nmol/mg protein). Lipid data were then scaled to the total molar content of all detected species in that lipid class. Compositional changes in lipid classes were subsequently evaluated by comparing individual species or groups of individual species (for acyl chain length and saturation profiles) with multiple paired *t* tests, controlling for a<10% false discovery rate (FDR) using the two-stage step-up method of Benjamini, Krieger, and Yekuieli (GraphPad Prism 9). Gene expression changes were assessed by multiple paired *t* tests controlling for FDR.

## Results

### Clinical Responses to Pioglitazone Treatment

**Figure 1:**
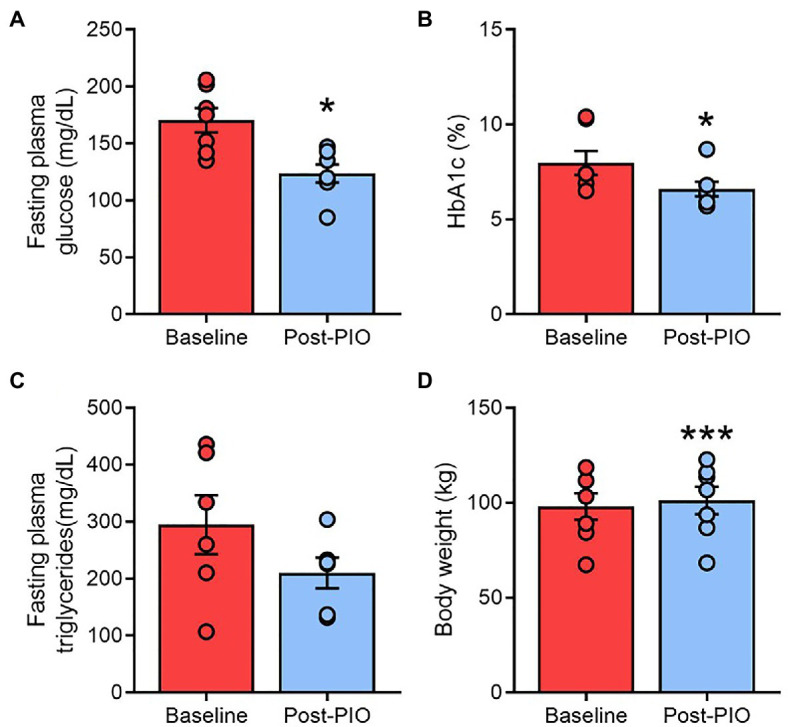
Clinical responses to pioglitazone treatment. Fasting plasma glucose **(A)**, glycated hemoglobin **(B)**, plasma triglycerides **(C)**, and body weight **(D)** at Baseline (red) and following 6months of 45mg/day pioglitazone treatment (blue). ^*^p<0.05, ^***^p<0.001 vs. Baseline. Data are mean±standard error (filled bars) and individual values (filled circles) for *n*=7 subjects.

In agreement with the established clinical effects of thiazolidinediones (Aronoff et al., 2000; Miyazaki et al., 2001), 6months of pioglitazone treatment lowered fasting blood glucose (Figure 1A) and HbA1c (Figure 1B), although the decline in fasting triglycerides (Figure 1C) did not reach statistical significance (*p*=0.085). Subjects gained an average of 3.2kg body weight following pioglitazone treatment (Figure 1D).

### Glycerophospholipid Profile Responds to Pioglitazone Treatment in Adipose Tissue

**Figure 2:**
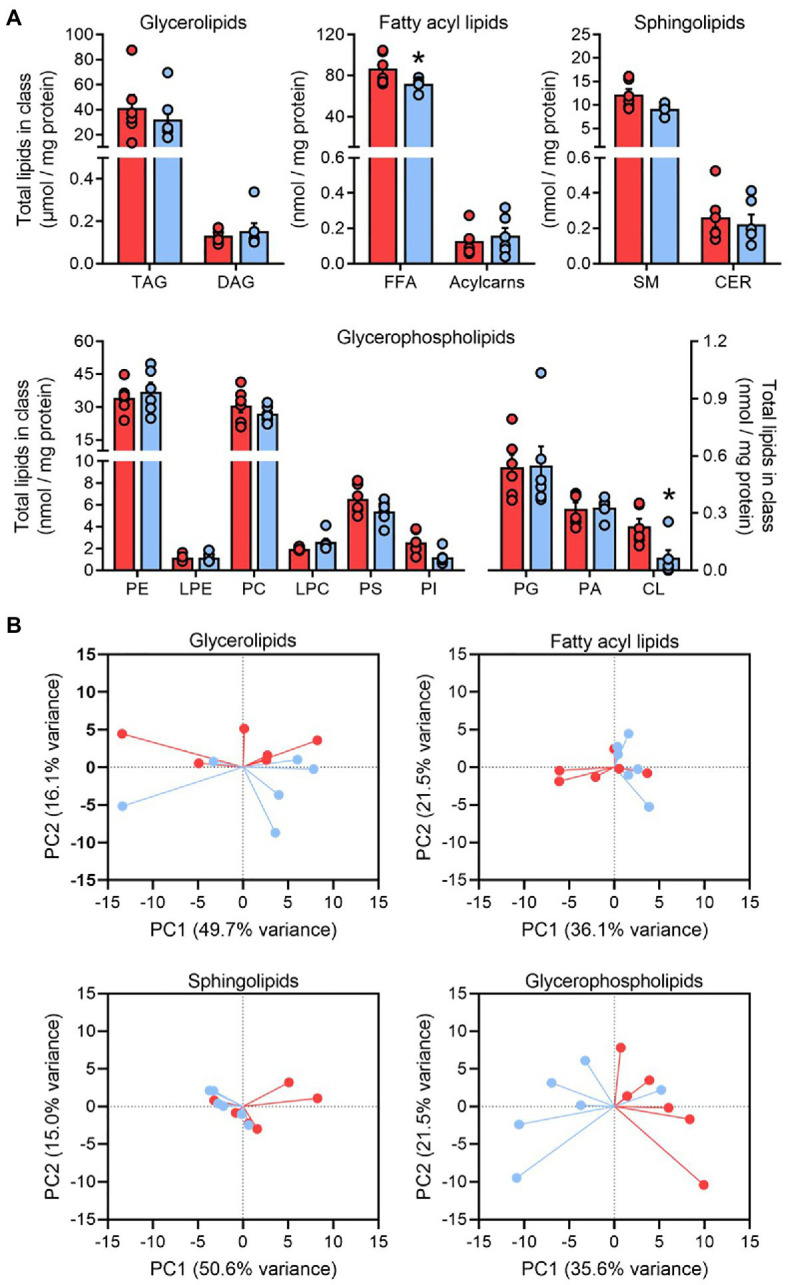
The glycerophospholipid profile responds to pioglitazone treatment in adipose tissue. Lipid class totals in adipose tissue **(A)** for triglyceride, diacylglyceride, free fatty acids, acylcarnitine (acylcarns), sphingomyelin (SM), ceramide, phosphatidylethanolamine (PE), lyso-phosphatidylethanolamine, phosphatidylcholine (PC), lyso-phosphatidylcholine, phosphatidylserine (PS), phosphatidylinositol (PI), phosphatidylglycerol (PG), phosphatidic acid (PA), and cardiolipin (CL) normalized to adipose protein content. ^*^p<0.05 vs. Baseline. Data are mean±standard error (filled bars) and individual values (filled circles) for *n*=6 subjects. Principal component analysis score plots of the first two principal components **(B)** summarizing the overall pattern of variance in glycerolipid, fatty acyl, sphingolipid, and glycerophospholipid pools from human adipose tissue at Baseline (red) and following 6months of 45mg/day pioglitazone treatment (blue). Note that pioglitazone-treated samples cluster separately along the x-axis (PC1) for glycerophospholipid scores.

Our class-targeted shotgun lipidomics approach determined the adipose tissue concentration of over 300 molecular lipid species across 15 functional lipid classes. In general, the total amount of each lipid class remained constant following pioglitazone treatment, apart from the free fatty acid and cardiolipin pools, which were both significantly decreased post treatment (Figure 2A). These observations were preserved regardless of whether lipid class totals were normalized to protein content or tissue weight (Supplementary Figure 1).

A wide inter-individual variability was observed for many lipid classes and, indeed, PCA of each major structural family of lipids (glycerolipids, glycerophospholipids, sphingolipids, and fatty acyl lipids) revealed that much of the variance in adipose lipid species was driven by between-subject differences (Figure 2B). However, baseline and post-pioglitazone samples clustered separately along PC1 for glycerophospholipids, with this vector explaining 36% of variance in all glycerophospholipid species (Figure 2B). This parallels previous observations that, in comparison with the dominant pool of adipose triglycerides, the composition of glycerophospholipids appears to be more responsive to changes in adipose tissue mass and/or metabolic function (Pietiläinen et al., 2011; May et al., 2017; Liu et al., 2020). As such, our subsequent analyses focused predominantly on the 141 measured lipid species comprising glycerophospholipid classes.

### Pioglitazone Increases the Saturation of Membrane Lipids in Adipose Tissue

**Figure 3:**
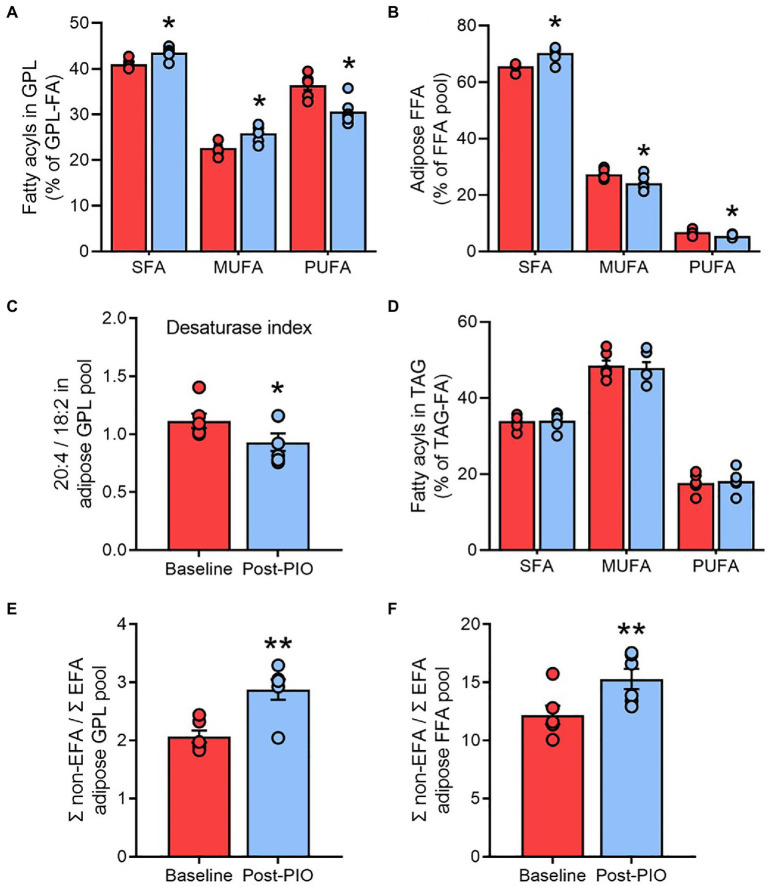
Pioglitazone increases the saturation of membrane lipids in adipose tissue. Contribution of saturated fatty acids, monounsaturated fatty acids, and polyunsaturated fatty acids to total fatty acyl groups in the glycerophospholipid **(A)**, free fatty acid **(B)**, and triglyceride **(D)** pools; ratio of 20:4 to 18:2 fatty acyl groups in glycerophospholipids as an index of fatty acid desaturase activity **(C)**; ratio of non-essential fatty acids (non-EFA) to essential fatty acids as an index of *de novo* lipogenesis in the glycerophospholipid **(E)** and free fatty acid **(F)** pools from adipose tissue at Baseline (red) and following 6months of 45mg/day pioglitazone treatment (blue). ^*^p<0.05, ^**^p<0.01 vs. Baseline. Data are mean±standard error (filled bars) and individual values (filled circles) for *n*=6 subjects.

Glycerophospholipids are critical components of cell membranes and the preferential trafficking of polyunsaturated fatty acids (PUFA) toward these complex lipids plays an important role in the regulation of membrane fluidity (Pietiläinen et al., 2011). Changes in the composition of glycerophospholipids, particularly their fatty acyl chain saturation, could therefore have important implications for adipocyte function. We considered the relative amounts of saturated fatty acids (SFA), monounsaturated fatty acids (MUFA), and PUFA in the total glycerophospholipid pool as an index of adipose cell membrane saturation. Pioglitazone treatment was associated with a significant decrease in the PUFA fraction, but an increase in the SFA fraction, in glycerophospholipids (Figure 3A). This shift toward a greater saturation of membrane lipids was similarly reflected in the free fatty acid pool (Figure 3B).

The fatty acid desaturases (FADS1 and FADS2) catalyze the rate-limiting enzymatic steps in the generation of PUFA, and an index of the combined flux through these pathways can be estimated from the product-to-substrate ratio of arachidonic acid to linoleic acid in tissue lipids (Martinelli et al., 2008; Del Pozo et al., 2020). Consistent with the increased saturation of membrane lipids, the FADS ratio calculated from the glycerophospholipid fatty acyl pool was significantly lower following pioglitazone treatment (Figure 3C), which may be indicative of a lower desaturase activity. However, no changes were observed in the saturation profile of the triglyceride pool (Figure 3D), which represents the overwhelming site of fatty acyl esterification in adipose tissue. These findings suggest that, consistent with our PCA plots (Figure 2B), the glycerophospholipid pool is highly responsive to pioglitazone-induced changes in unsaturated free fatty acid availability, whereas the triglyceride pool is more resistant to these changes.

Another process closely linked to fatty acid desaturation is *de novo* lipogenesis (DNL), which can be similarly estimated from the ratio of non-essential fatty acids (which can be synthesized) to essential fatty acids (obtained exclusively from the diet) in the glycerophospholipid pool (Yew Tan et al., 2015). In agreement with previous reports that thiazolidinediones enhance DNL in human adipose tissue (de Souza et al., 2001; McTernan et al., 2002), the DNL index was significantly increased by pioglitazone treatment (Figure 3E), a finding that was again replicated in the free fatty acyl pool (Figure 3F). Moreover, these observations were substantiated by another common metric of DNL, the palmitate (C16:0) to linoleate (C18:2) ratio, in both the glycerophospholipid (Supplementary Figure 2A) and free fatty acid pools (Supplementary Figure 2B).

### Pioglitazone Lowers Arachidonic Acid Enrichment in Adipose Glycerophospholipids

**Figure 4:**
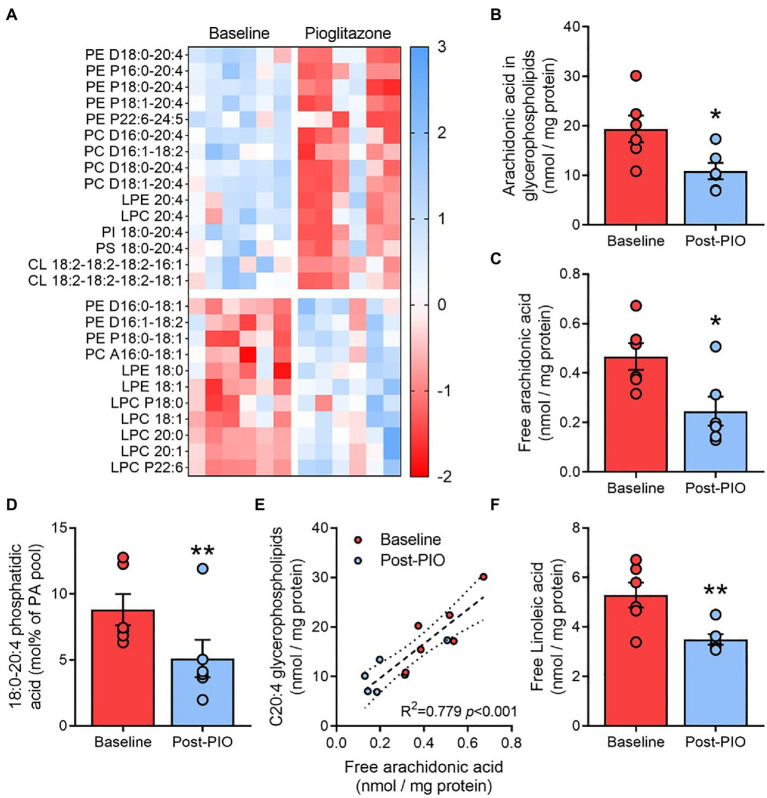
Pioglitazone lowers arachidonic acid enrichment in adipose glycerophospholipids. Heatmap depicting fold change (*z* scores) for significantly altered molecular glycerophospholipid species in paired adipose tissue samples **(A)**, with blue indicating an upregulation and red indicating a downregulation following 6months of 45mg/day pioglitazone treatment. Lipids were first normalized to total glycerophospholipid content detected in each respective sample and compared with multiple paired *t* tests corrected for a false discovery rate of 10%. Absolute content of arachidonic acid esterified in glycerophospholipids **(B)** and in unesterified form **(C)**, and its fractional contribution in phosphatidic acid **(D)**, as well as the correlation between free and glycerophospholipid esterified arachidonic acid **(E)** and absolute content of linoleic acid **(F)** in paired adipose tissue samples at Baseline (red) and Post-PIO (blue). ^*^p<0.05, ^**^p<0.01 vs. Baseline. Data are mean±standard error (filled bars) and individual values (filled circles) for *n*=6 subjects.

To ascertain whether certain functional lipid groups or species were responsible for the observed changes in membrane lipid saturation following pioglitazone treatment, each molecular species was next normalized to the respective total glycerophospholipid content determined in each sample. After controlling for multiple comparisons (see *Statistical Analyses* in Methods), 26 species (15 downregulated, 11 upregulated) were found to be altered by pioglitazone treatment (Figure 4A). Strikingly, two-thirds of downregulated glycerophospholipids were species containing the long-chain polyunsaturated fatty acid arachidonic (C20:4) acid (AA). Indeed, the cumulative amount of AA esterified in glycerophospholipids was 40% lower following pioglitazone treatment (Figure 4B), while AA-containing species also accounted for many of the strongest positive loadings on PC1 for glycerophospholipids (Supplementary Figure 3A). In contrast, no change was observed in the glycerophospholipid levels of docosahexaenoic acid (C22:6; Supplementary Figure 3B), another major long-chain polyunsaturated fatty acid. Moreover, the AA content of adipose triglycerides also remained unchanged following pioglitazone treatment (Supplementary Figure 3C). Together, these findings highlight a selective decrease in glycerophospholipid AA enrichment as a key feature of the molecular lipid response to pioglitazone treatment in adipose tissue.

Changes in the arachidonic acid content of glycerophospholipids could be related to alterations in free arachidonic acid availability and its subsequent esterification into phosphatidic acid, the obligate precursor for all glycerophospholipids. Accordingly, pioglitazone treatment resulted in a~45% reduction in free AA availability (Figure 4C), which was paralleled by a decrease in 18:0–20:4 phosphatidic acid (Figure 4D), the major AA-containing phosphatidic acid species. Moreover, free AA concentrations were positively and significantly correlated with the AA content of glycerophospholipids across all samples and timepoints (Figure 4E). The cellular pool of free AA is partially dependent upon its synthesis from the essential fatty acid linoleic acid (Hanna and Hafez, 2018). Adipose tissue levels of free linoleic acid were also significantly reduced after pioglitazone treatment (Figure 4F), supporting a decrease in AA synthesis as a possible mechanism through which pioglitazone influences glycerophospholipid remodeling.

### Adipose Tissue Plasmenylethanolamines Are Lowered by Pioglitazone Treatment

**Figure 5:**
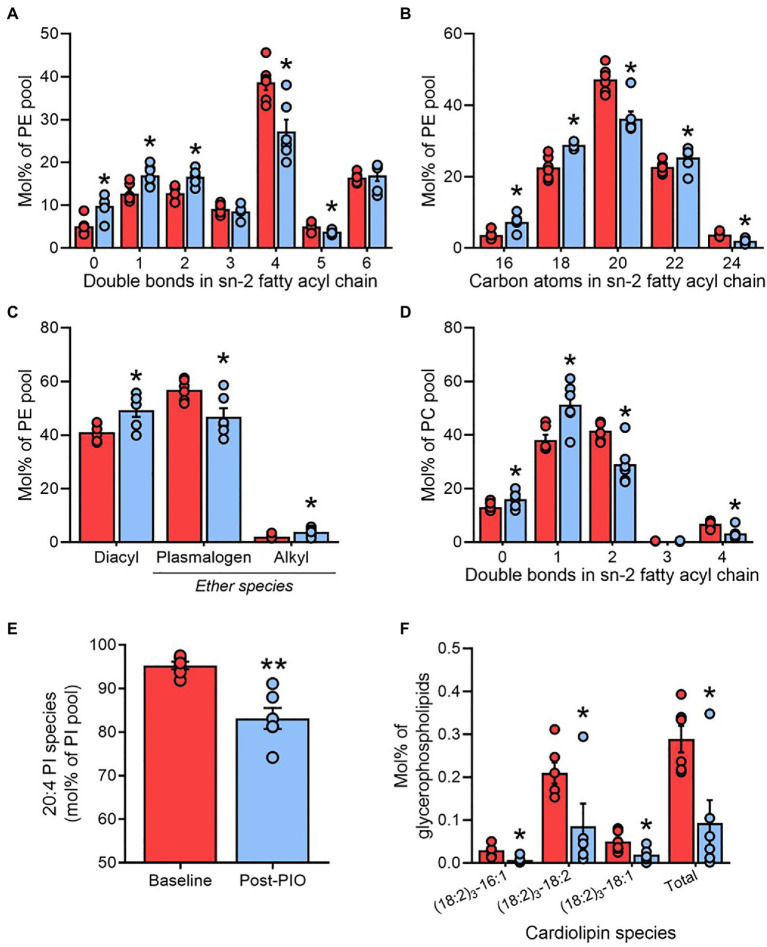
Adipose tissue plasmenylethanolamines are lowered by pioglitazone treatment. Saturation number of double bonds; **(A)** and length number of carbon atoms; **(B)** of fatty acyl chains in the *sn-2* position, and ether chain content **(C)**, of phosphatidylethanolamine (PE); Saturation of fatty acyl chains in the *sn-2* position of phosphatidylcholine **(D)**; arachidonic acid enrichment in phosphatidylethanolamine **(E)**; content of cardiolipins **(F)** in paired adipose tissue samples at Baseline (red) and Post-PIO (blue). ^*^p<0.05, ^**^p<0.01 vs. Baseline. Data are mean±standard error (filled bars) and individual values (filled circles), normalized to the total molar content for each respective lipid class **(A–E)**, or to the total glycerophospholipid pool **(F)**, for *n*=6 subjects.

Phosphatidylethanolamines (PE) are the most abundant glycerophospholipids in adipose tissue (Figure 2A) and represent the major site of AA accumulation in most mammalian cell membranes, specifically at the *sn-2* fatty acyl position. Consistent with the profile changes of total glycerophospholipids (Figure 3A), pioglitazone treatment was associated with an increased saturation of the *sn-2* fatty acyl chain in PE (Figure 5A), as well as replacement of fatty acyl species containing 20 carbons by predominantly shorter (C16 and C18) species (Figure 5B). In contrast, the composition of the fatty acyl chain in the *sn-1* position of PE, which does not accrue AA, was unaltered by pioglitazone treatment (Supplementary Figures 4A,B).

Compared with other glycerophospholipid classes, PE contain a greater portion of plasmalogen species (plasmenylet hanolamines), which are especially highly enriched in AA (Brites et al., 2004) and have previously been associated with adipose tissue expansion in human obesity (Pietiläinen et al., 2011). Numerous plasmenylethanolamines were altered by pioglitazone (Figure 4A), culminating in a decrease in the contribution of plasmalogens to the total PE pool relative to diacyl and alkyl forms (Figure 5C). A more modest pattern of increased acyl chain saturation was reflected in the *sn-2* position of phosphatidylcholine (PC), another major constituent of cellular membranes, consistent with the lower enrichment of AA in PC vs. PE (Figure 5D). The increased remodeling of cell membrane lipids was further reflected by alterations in the lyso-phospholipid pool. Whereas the relative levels of 20:4 lyso-PE and lyso-PC were reduced following pioglitazone treatment, many other (non-20:4) species were upregulated (Figure 4A).

### Pioglitazone Influences Lipid Mediators of Insulin Signaling and Mitochondria in Adipose Tissue

Another group of lipids tightly linked to AA metabolism is the phosphatidylinositols (PI; Anderson et al., 2016), serving as a precursor pool for the downstream generation of phosphatidyl 3,4,5-triphosphate (PIP_3_) and therefore playing a vital role in the regulation intracellular insulin signaling (Sandra and Marshall, 1986). In agreement with conserved observations across mammalian cell types (Patton et al., 1982; Kurvinen et al., 2000; Wang et al., 2016), PI 18:0–20:4 predominated as the major molecular PI species in adipose tissue (Supplementary Figure 4C). However, consistent with the reduction in AA availability (Figure 4C), PI species containing 20:4 acyl moieties were markedly lower following pioglitazone treatment (Figure 5E and Supplementary Figure 4C), whereas the contribution of other species to the total PI pool increased (Supplementary Figure 4C).

As well as contributing to AA synthesis, linoleic acid is a crucial component of the mitochondrial membrane lipid cardiolipin. Consistent with the lower mitochondrial content in human white adipose tissue, total cardiolipin concentrations detected in adipose tissue were nearly 10-fold lower than that measured in skeletal muscle from the same subjects (Figure 2A and Supplementary Figure 4D). As previously acknowledged, and in agreement with our previous findings in livers from pioglitazone-treated mice (Shannon et al., 2021), adipose tissue cardiolipin was significantly reduced by pioglitazone treatment (Figure 2A). This decrease was not driven by changes in particular molecular species of cardiolipin but was instead related to a universal reduction in all detected species (Figure 5F).

### Pioglitazone-Induced Changes in Adipose Tissue Are Partially Recapitulated in Skeletal Muscle

**Figure 6:**
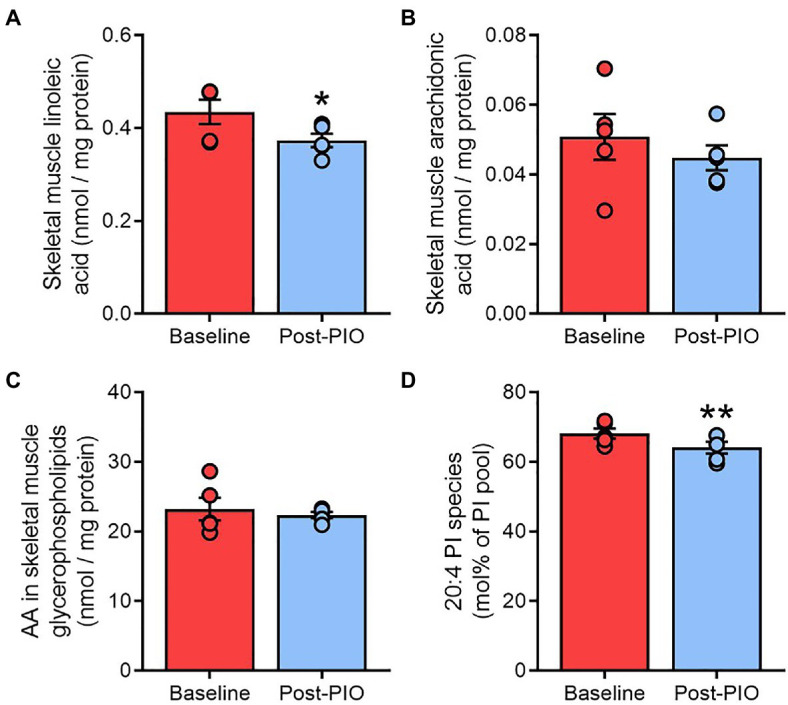
Pioglitazone-induced changes in adipose tissue are partially recapitulated in skeletal muscle. Content of free linoleic acid **(A)** and arachidonic acid in unesterified form **(B)** or esterified in glycerophospholipids **(C)**, or as a fraction of the phosphatidylinositol pool **(D)**, in paired skeletal muscle samples at Baseline (red) and Post-PIO (blue). ^*^p<0.05, ^**^p<0.01 vs. Baseline. Data are mean±standard error (filled bars) and individual values (filled circles) for *n*=5 subjects.

Skeletal muscle is another major tissue targeted by the insulin-sensitizing effects of pioglitazone (Bajaj et al., 2010). We evaluated whether the pioglitazone-induced changes observed in adipose tissue were also evident in skeletal muscle biopsies from a subset (*n*=5) of the same subjects. Free linoleic acid concentrations in skeletal muscle were~15% lower following pioglitazone treatment (Figure 6A), although this was not associated with alterations in either the level of free AA (Figure 6B) or its enrichment in the total glycerophospholipid pool (Figure 6C). Nevertheless, AA enrichment in phosphatidylinositol declined significantly with pioglitazone (Figure 6D). Thus, part of the lipid remodeling effects of pioglitazone observed in human adipose tissue are paralleled by changes in skeletal muscle.

### Impact of Pioglitazone on Phospholipase Gene Expression

**Figure 7:**
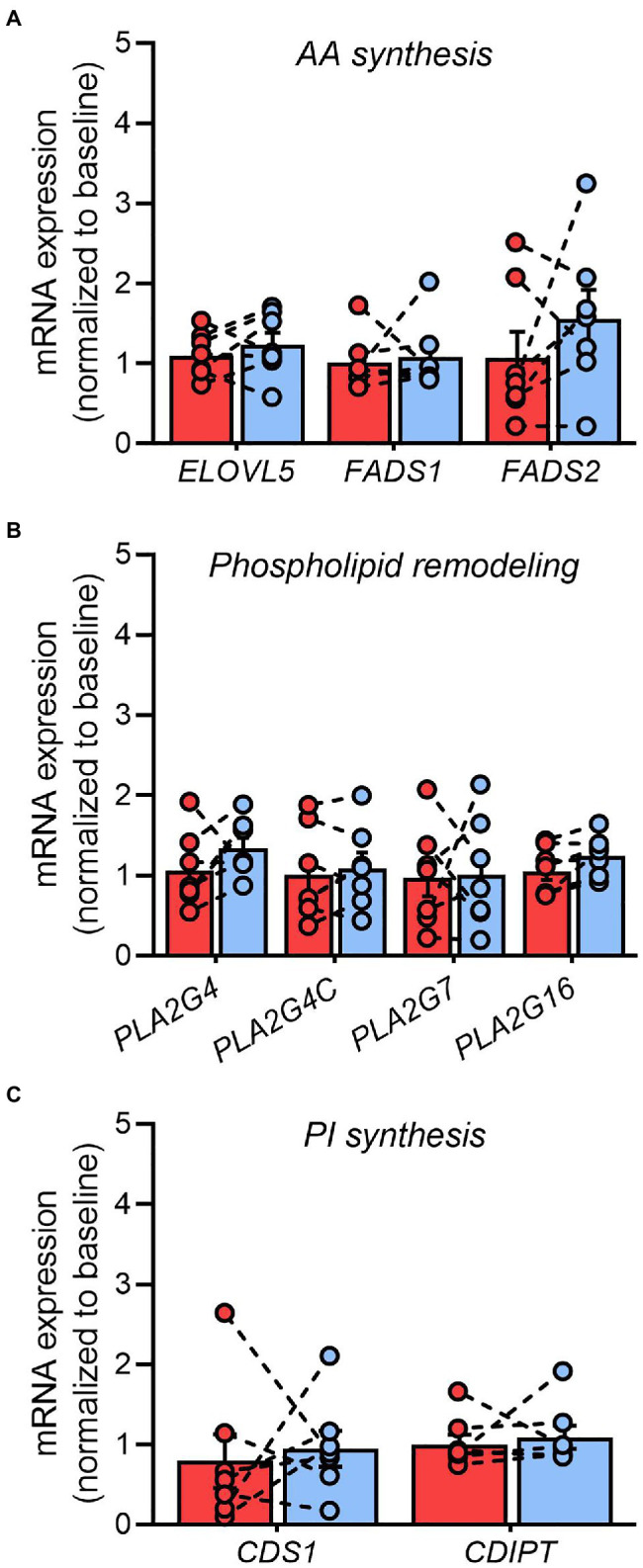
Pioglitazone does not influence phospholipase gene expression in adipose tissue. Fold change in the adipose tissue mRNA expression of genes involved in arachidonic acid synthesis **(A)**, glycerophospholipid hydrolysis **(B)**, and phosphatidylinositol synthesis **(C)** following 6months of 45mg/day pioglitazone treatment. Target genes were first normalized to the geometric mean of *ACTB* and *GAPDH* and subsequently normalized to the average of Baseline samples. *ELOVL5* (fatty acid elongase 5), *FADS1* (fatty acid desaturase 1), *FADS2* (fatty acid desaturase 2), *PLA2G4* (cytosolic phospholipase A2), *PLA2G4C* (cytosolic lysophospholipase), *PLA2G7* (lipoprotein-associated phospholipase A2), *PLA2G16* (adipose-specific phospholipase A2), *CDS1* (CDP-diacylglycerol synthase 1), *CDIPT* (CDP-diacylglycerol-inositol-3-phosphatidyltransferase/phosphatidylinositol synthase). Data are mean±standard error (filled bars) and individual values (filled circles) for *n*=7 subjects.

Since many of the disease-modifying actions of pioglitazone have been attributed to transcriptional modulation of lipid metabolism pathways (Spiegelman, 1998), we explored prospective changes in genes involved in the regulation of AA metabolism. The adipose tissue mRNA expression of genes involved in AA synthesis from linoleic acid (*ELOVL5*, *FADS1*, and *FADS2*; Figure 7A), or AA removal from glycerophospholipids (*PLA2G4*, *PLA2G4B*, *PLA2G4C*, *PLA2G7*, and *PLA2G16*; Figure 7B), remained unchanged after 6months of pioglitazone treatment. Similarly, no differences were observed in the genes responsible for AA incorporation into phosphatidylinositol, including *CDS1* or *CDIPT* (Figure 7C).

## Discussion

Despite being prescribed as an anti-diabetic agent for over 20years, the precise molecular mechanisms of pioglitazone remain unresolved. The results of the current study demonstrate that the clinical benefits seen in T2D patients treated with pioglitazone are accompanied by robust compositional changes in adipose tissue glycerophospholipids, with minimal alterations observed in other lipid classes. Specifically, pioglitazone-induced adipose remodeling was characterized by an increased saturation of membrane lipids, driven primarily by reductions in glycerophospholipid species enriched for arachidonic acid (AA), including phosphatidylinositols and plasmenylethanolamines. These changes were not dependent upon transcriptional activation of phospholipase genes but were associated with a decrease in adipose tissue levels of free AA and its parent precursor linoleic acid. Notably, despite weight gain, which is likely indicative of an expansion of the subcutaneous adipose tissue depots (Bray et al., 2013), the molecular profile of lipid storage in adipose triglycerides was unchanged by pioglitazone, suggesting a targeted remodeling of lipids within cellular membranes. These data provide evidence that the (mal)adaptive remodeling of adipose tissue glycerophospholipids observed in human obesity (Pietiläinen et al., 2011; Liu et al., 2020) is reversible by insulin-sensitizing therapy and, moreover, identify AA metabolism as a central node in this process.

Pioglitazone is classically understood to improve insulin sensitivity and cardiometabolic health by reversing lipotoxicity in muscle and liver, second to increasing lipid storage in adipose tissue. The results from our current study offer support for this paradigm. Indeed, pioglitazone caused a shortening and increased saturation of fatty acyl chains in glycerophospholipids, a trait that was recently shown to characterize the differentiation of preadipocytes into mature adipocytes, at least for *in vitro* models of adipogenesis (Miehle et al., 2020). Moreover, pioglitazone treatment increased indices of DNL, which is another demonstrable feature of smaller, more insulin sensitive adipocytes (Roberts et al., 2009). By contrast, the enlargement of existing adipocytes under obesogenic conditions (i.e., hypertrophy) is associated with a more elongated, unsaturated fatty acyl profile in adipose tissue, particularly for membrane lipids (Pietiläinen et al., 2011; Yew Tan et al., 2015), as well as a downregulation of DNL (Roberts et al., 2009). The changes in membrane lipids observed here may thus reflect a reversal of obesity-associated processes and are consistent with a pioglitazone-mediated *de novo* formation of smaller adipocytes (McLaughlin et al., 2010).

Studies in both rodents (Grzybek et al., 2019; Liu et al., 2020) and humans (Pietiläinen et al., 2011) have reported an obesity-associated decrease in the fatty acyl saturation of adipose tissue glycerophospholipids. This remodeling of cell membrane lipids is believed to be necessary to accommodate the sustained expansion of adipose tissue depots with progressive weight gain. One prior lipidomic investigation of twin pairs discordant for obesity identified an increase in polyunsaturated PEs, specifically plasmenylethanolamine species, as an important feature of this adipose remodeling (Pietiläinen et al., 2011). Supported by computer simulations, the authors speculated that the increase in vinyl-ether bonds present in plasmalogens (which decrease membrane fluidity) served to maintain healthy cell membrane function despite the opposing effects of glycerophospholipid desaturation (which increases membrane fluidity). Reciprocally, therefore, the selective reduction in plasmenylethanolamines following pioglitazone treatment could be expected to offset potential alterations in membrane fluidity that might otherwise accompany the increased saturation of glycerophospholipids. Although we did not measure adipose tissue mass in the current study, the observed weight gain was comparable to previous studies in which increased adiposity with pioglitazone was directly demonstrated by dual X-ray absorptiometry (Bray et al., 2013). Our findings thus provide evidence that the parallel expansions of adipose tissue with obesity vs. pioglitazone are likely associated with opposing effects on membrane lipid remodeling.

A high content of plasmalogen lipids in the adipose tissue of obese individuals has been implicated in the activation of inflammatory pathways (Pietiläinen et al., 2011) since these species are particularly vulnerable to oxidative stress (Scherrer and Gross, 1989). As such, the observed reduction in plasmenylethanolamines may also represent an important, novel mechanism through which pioglitazone treatment corrects adipose dysfunction to lower systemic inflammation in obesity and T2D (DeFronzo, 2004). The decrease in AA, a crucial component of plasmenylethanolamines, may be especially pertinent to the therapeutic actions of pioglitazone in adipose tissue. Indeed, adipose tissue levels of AA are reportedly increased in obesity (Williams et al., 2007; Pietiläinen et al., 2011) and this omega-6 PUFA, as well as its lipid derivatives, has been shown to activate pro-inflammatory pathways (Schreiber and Zechner, 2014) and antagonize insulin-mediated glucose uptake (Tebbey et al., 1994) in adipocytes. By contrast, the omega-3 PUFA DHA, which was unchanged by pioglitazone, is believed to promote anti-inflammatory signaling (Kuda et al., 2016). As such, the shift toward a lower adipose AA/DHA ratio following pioglitazone treatment likely represents a more metabolically protective PUFA profile (Pietiläinen et al., 2011).

The selective decrease in AA-containing glycerophospholipids with pioglitazone, including plasmenylethanolamines, could result from either an increase in glycerophospholipid turnover (i.e., hydrolysis) or from the suppression of AA synthesis and/or esterification. In alignment with the former possibility, one previous study demonstrated that acute incubation of fibroblasts with pioglitazone accelerated the release of AA from cell membranes (Tsukamoto et al., 2004). Moreover, and in agreement with the current findings, this was not mediated by transcriptional or post-translational regulation of phospholipase activity but was instead attributed to a pioglitazone-mediated inhibition of AA reuptake. However, chronic blockade of membrane AA reuptake over 6 months of pioglitazone treatment would be expected to promote the accumulation of free AA or at least its diversion into other pathways. On the contrary, adipose tissue AA availability was lower following pioglitazone treatment and was tightly correlated with the decrease in glycerophospholipid AA esterification. Similarly, 20:4 lyso-PE, a product of the partial de-acylation of AA-containing PE species, was also lower following pioglitazone. Taken together with the decline in 18:0–20:4 phosphatidic acid, these findings suggest that the reduction in glycerophospholipid AA content, and subsequent suppression of plasmenylethanolamine concentrations, was likely related to a reduction in the partitioning of AA into glycerophospholipids rather than an increase in their turnover.

Another interesting finding from the current study was that levels of linoleic acid were reduced following pioglitazone treatment, both in adipose and skeletal muscle tissues. Since linoleic acid is an obligate precursor for the synthesis of other omega-6 PUFAs, it seems likely that its reduction contributed to the decrease in AA available for esterification into glycerophospholipids. Notably, the lower linoleic acid level was also mirrored by a significant reduction in adipose tissue cardiolipins, a mitochondrial membrane lipid highly enriched in linoleoyl side chains. Cardiolipin is often used as a marker of mitochondrial mass and, as such, this finding appears to contradict previous reports that thiazolidinediones increase mitochondrial content and respiratory complex expression in human adipose tissue (Bogacka et al., 2005; Xie et al., 2017). However, considering that our lipidomics analysis was performed on bulk adipose tissue and that white adipocytes are relatively poor in mitochondria (Cedikova et al., 2016), while infiltrating immune cells are relatively rich in mitochondria (e.g., lymphocytes; Picard, 2021) and activated macrophages possess higher mitochondrial mass than resting cells (Yu et al., 2020), the observed differences in cardiolipin content could potentially reflect differences in the cellular makeup of the adipose tissue. Indeed, previous studies have demonstrated a reduction in human adipose tissue inflammation following pioglitazone treatment through reduction of macrophages and mast cells (Spencer et al., 2014). Thus, the observed reduction in total cardiolipin levels after pioglitazone treatment may reflect fewer infiltrating macrophages. Moreover, we recently reported that pioglitazone treatment reduced hepatic cardiolipin content in obese mice and was associated with a suppression of mitochondrial fluxes (Shannon et al., 2021). Accumulating evidence suggests that pathological cardiolipin remodeling characterizes conditions of high oxidative stress, including diabetic cardiomyopathy (Han et al., 2007) and non-alcoholic fatty liver disease (Wang et al., 2015). This remodeling is typified by an increased abundance of long acyl chain cardiolipin species and was reversed by pioglitazone treatment in our previous study (Shannon et al., 2021). The low abundance of mitochondria in adipose tissue precluded the detection of these long acyl chain cardiolipin species in the current study and may thus have masked patterns of cardiolipin remodeling following pioglitazone treatment. As such, future studies are needed to reconcile discrepancies between cardiolipin profiles and the mitochondrial effects of pioglitazone treatment in adipose tissue.

The mechanism through which pioglitazone treatment lowered tissue linoleic acid is unclear. Since we did not monitor dietary patterns in the current study, we cannot exclude the possibility that the intake of linoleic acid, an essential fatty acid, declined over the six-month intervention period. However, alterations in dietary linoleic acid do not appear to influence the systemic availability of either linoleic acid or arachidonic acid (Rett and Whelan, 2011), suggesting that a decrease in linoleic acid intake is unlikely to be responsible for the lower tissue concentrations of linoleic acid following pioglitazone treatment. A more likely explanation for the decline in both linoleic acid and AA concentrations involves the activation of PPARγ, which is the primary molecular target of thiazolidinediones in adipose tissue (Spiegelman, 1998). Indeed, PPARγ agonism by thiazolidinediones is known to upregulate both lipid storage and lipid oxidation pathways in adipose tissue of T2D patients (Boden et al., 2005). Additionally, since AA and its derivatives can act as natural ligands for PPARγ (Nikolopoulou et al., 2014), it is feasible that a negative feedback loop exists coupling a reduced production of endogenous ligand metabolites with the sustained activation of PPARγ by exogenous ligands such as pioglitazone.

It should be acknowledged that the reduction in PUFA-containing lipids could be indicative of their selective metabolism by peroxisomal β-oxidation, since the master driver of peroxisomes PPARα (Lee et al., 1995) has been implicated in the therapeutic efficacy of pioglitazone (Orasanu et al., 2008). However, peroxisomes serve as the exclusive site of plasmalogen synthesis (Thai et al., 2001) and, therefore, their putative upregulation by pioglitazone cannot explain the observed reduction in plasmalogens following pioglitazone treatment. Indeed, while the effects of PPARα agonists in human adipose tissue are poorly defined, it is notable that fenofibrates do not influence peroxisomal density (size or number) in human liver (Gariot et al., 1983). Moreover, human studies dissecting the divergent effects of pioglitazone and fenofibrate (monotherapy and combination) revealed that improvements in insulin sensitivity, glycemic control, and lipid metabolism following pioglitazone are near-exclusively driven by PPARγ and not PPARα (Bajaj et al., 2007; Belfort et al., 2010).

A final noteworthy effect of pioglitazone treatment related to AA metabolism was the reduction in AA-containing phosphatidylinositol. Although this effect was more robust in adipose tissue, it was also observed in skeletal muscle and is consistent with *in vitro* evidence that free AA availability can influence its enrichment in phosphatidylinositol (Anderson et al., 2016). At present, very little is known about how the acyl chain composition of phosphatidylinositol impacts upon its function. However, given the integral role of phosphatidylinositol derivatives (e.g., polyphosphoinositides PIP_2_ and PIP_3_) in the insulin signaling pathway (Barneda et al., 2019), our data raise the intriguing possibility that the molecular link between AA availability and phosphatidylinositol remodeling may contribute to the insulin-sensitizing effects of pioglitazone in peripheral tissues.

In summary, our study provides the first lipidomic characterization of the chronic effects of pioglitazone on adipose tissue in humans. The findings identify the glycerophospholipid pool as a central transducer of the responses to pioglitazone treatment, highlighting a potential role for adipose cell membrane remodeling in the immunometabolic benefits of thiazolidinediones. Our results derive from a relatively small and mostly (86%) male sample and thus warrant further validation in expanded populations, especially given the increasingly appreciated role of sexual dimorphism in adipose tissue metabolism and health (MacCannell et al., 2021; Pan and Chen, 2021). The lack of a control group is also a shortcoming, although it is partially compensated by assessment of the lipidome at baseline (pre-treatment). Nevertheless, our findings are consistent with known physiological effects of pioglitazone that have been robustly demonstrated in larger human studies (Miyazaki et al., 2002; Bray et al., 2013; White et al., 2021). Overall, our data support the targeting of pathways involved in the regulation of adipocyte cell membrane function as a novel approach to combat the metabolic and inflammatory sequelae of obesity and T2D.

## Data Availability Statement

The raw data supporting the conclusions of this article will be made available by the authors, without undue reservation.

## Ethics Statement

The studies involving human participants were reviewed and approved by Institutional Review Board of the South Texas Veterans Healthcare System, University of Texas health Science Center San Antonio. The patients/participants provided their written informed consent to participate in this study.

## Author Contributions

All authors contributed to the data analysis and interpretation of the data, drafting, and revising the manuscript and approved the final version of the manuscript. The study design was conceptualized by JP, DT, RD, and CS. Methodology was performed by JP, AC-V, DT, MP, and MF. Funding acquisition, RD and CS.

## Funding

CS is supported by an RL5 training scholarship from the San Antonio Claude D. Pepper Older Americans Independence Center.

## Conflict of Interest

The authors declare that the research was conducted in the absence of any commercial or financial relationships that could be construed as a potential conflict of interest.

## Publisher’s Note

All claims expressed in this article are solely those of the authors and do not necessarily represent those of their affiliated organizations, or those of the publisher, the editors and the reviewers. Any product that may be evaluated in this article, or claim that may be made by its manufacturer, is not guaranteed or endorsed by the publisher.

## Supplementary Material

Supplementary Figure 1 Lipid class totals in adipose tissue for triglyceride (TAG), diacylglyceride (DAG), free fatty acids (FFA), acylcarnitine (acylcarns), sphingomyelin (SM), ceramide (CER), phosphatidylethanolamine (PE), lyso-phosphatidylethanolamine (LPE), phosphatidylcholine (PC), lyso-phosphatidylcholine (LPC), phosphatidylserine (PS), phosphatidylinositol (PI), phosphatidylglycerol (PG), phosphatidic acid (PA), and cardiolipin (CL) normalized to adipose tissue wet weight. ^*^*p*<0.05 versus Baseline. Data are mean±standard error (filled bars) and individual values (filled circles) for *n*=6 subjects.

Supplementary Figure 2 Adipose tissue ratio of C16:0 (palmitate)/C18:2 (linoleate) in glycerophospholipids **(A)** and free fatty acids **(B)** as an index of *de novo* lipogenesis. ^*^*p*<0.05 versus Baseline. Data are mean±standard error (filled bars) and individual values (filled circles) for *n*=6 subjects.

Supplementary Figure 3 Strongest (top 15%) loadings from the first principal component of glycerophospholipids **(A)** which differentiated Baseline from Post-PIO adipose samples. Positive loadings (downregulated by pioglitazone) are in red and negative loadings (upregulated by pioglitazone) are in blue. Loadings which identify an arachidonic acid (20:4)-containing glycerophospholipid species are in bold text. Adipose tissue content of docosahexaenoic acid (C22:6) in glycerophospholipids **(B)** and arachidonic acid (C20:4) in triglycerides **(C)**. Data are mean ± standard error (filled bars) and individual values (filled circles) for *n*=6 subjects.

Supplementary Figure 4 Saturation [number of double bonds; **(A)**] and length (number of carbon atoms; **(B)** of fatty acyl chains in the *sn-1* position of phosphatidylethanolamine; Fatty acyl composition of phosphatidylinositol **(C)** in adipose tissue, expressed as a molar fraction of total lipids in respective class. Skeletal muscle total cardiolipin content **(D)**. Data are mean±standard error (filled bars) and individual values (filled circles) for *n*=6 **(A–C)** or *n*=5 **(D)** subjects.
