# Supplementary figures and images for: The Insulin-Sensitizer Pioglitazone Remodels Adipose Tissue Phospholipids in Humans
Source: Front Physiol. 2021 Dec 2;12:784391. doi: 10.3389/fphys.2021.784391 (PMC8674727; doi:10.3389/fphys.2021.784391)

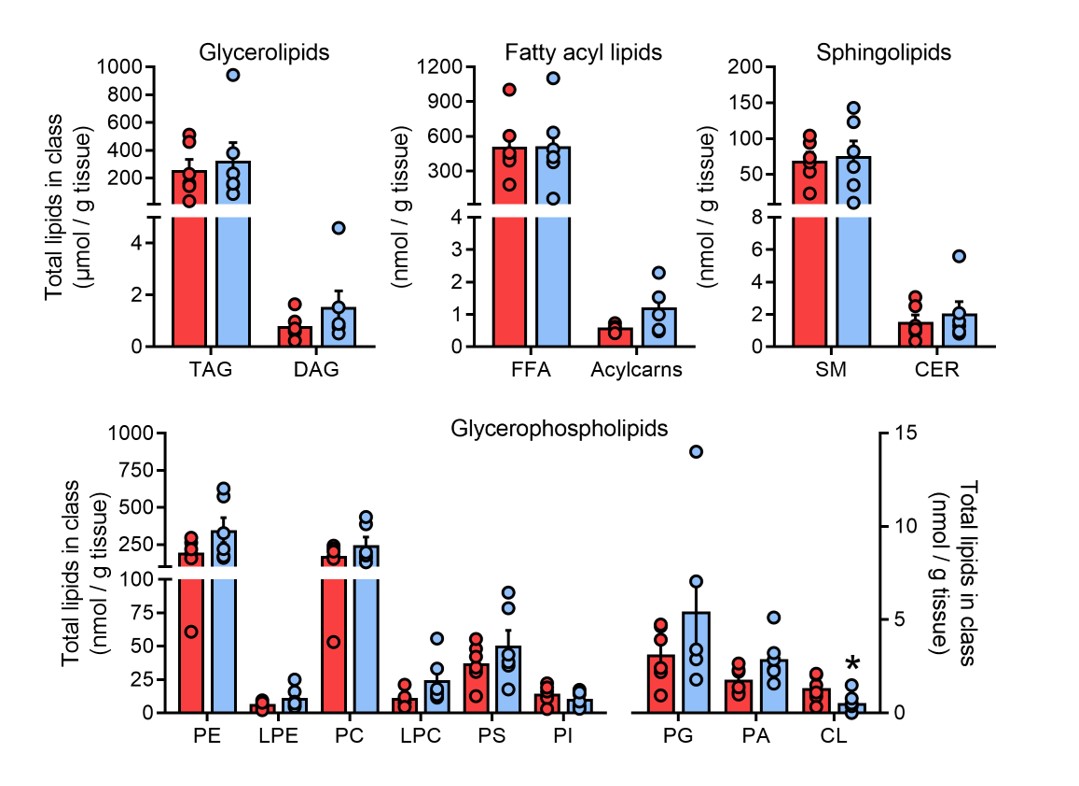

Supplement: Supplementary Figure 1 — Lipid class totals in adipose tissue for triglyceride (TAG), diacylglyceride (DAG), free fatty acids (FFA), acylcarnitine (acylcarns), sphingomyelin (SM), ceramide (CER), phosphatidylethanolamine (PE), lyso-phosphatidylethanolamine (LPE), phosphatidylcholine (PC), lyso-phosphatidylcholine (LPC), phosphatidylserine (PS), phosphatidylinositol (PI), phosphatidylglycerol (PG), phosphatidic acid (PA), and cardiolipin (CL) normalized to adipose tissue wet weight. *p<0.05 versus Baseline. Data are mean±standard error (filled bars) and individual values (filled circles) for n=6 subjects. [file Image_1.JPEG]

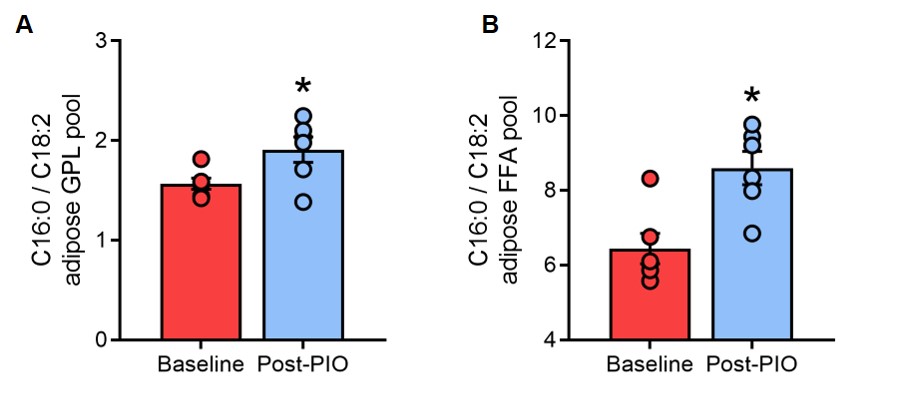

Supplement: Supplementary Figure 2 — Adipose tissue ratio of C16:0 (palmitate)/C18:2 (linoleate) in glycerophospholipids (A) and free fatty acids (B) as an index of de novo lipogenesis. *p<0.05 versus Baseline. Data are mean±standard error (filled bars) and individual values (filled circles) for n=6 subjects. [file Image_2.JPEG]

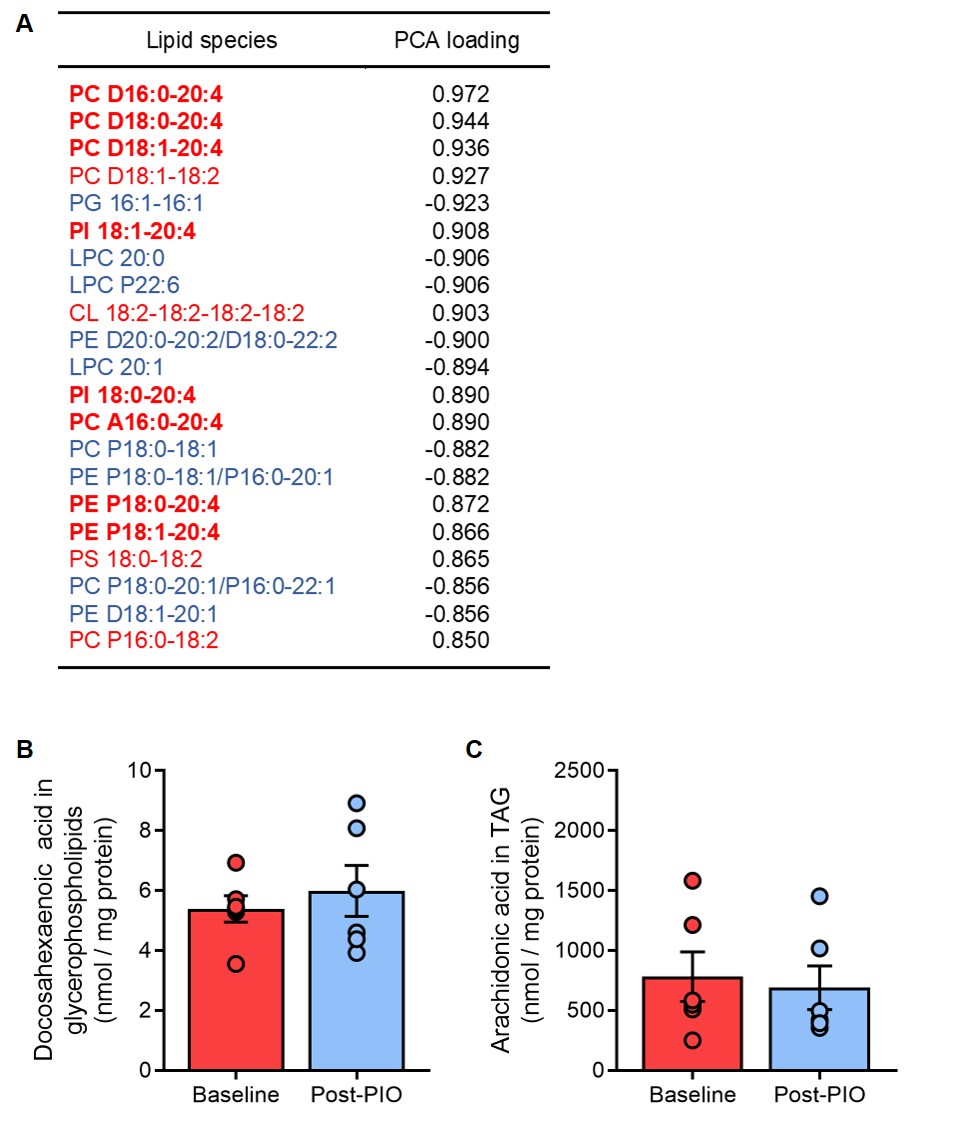

Supplement: Supplementary Figure 3 — Strongest (top 15%) loadings from the first principal component of glycerophospholipids (A) which differentiated Baseline from Post-PIO adipose samples. Positive loadings (downregulated by pioglitazone) are in red and negative loadings (upregulated by pioglitazone) are in blue. Loadings which identify an arachidonic acid (20:4)-containing glycerophospholipid species are in bold text. Adipose tissue content of docosahexaenoic acid (C22:6) in glycerophospholipids (B) and arachidonic acid (C20:4) in triglycerides (C). Data are mean ± standard error (filled bars) and individual values (filled circles) for n=6 subjects. [file Image_3.JPEG]

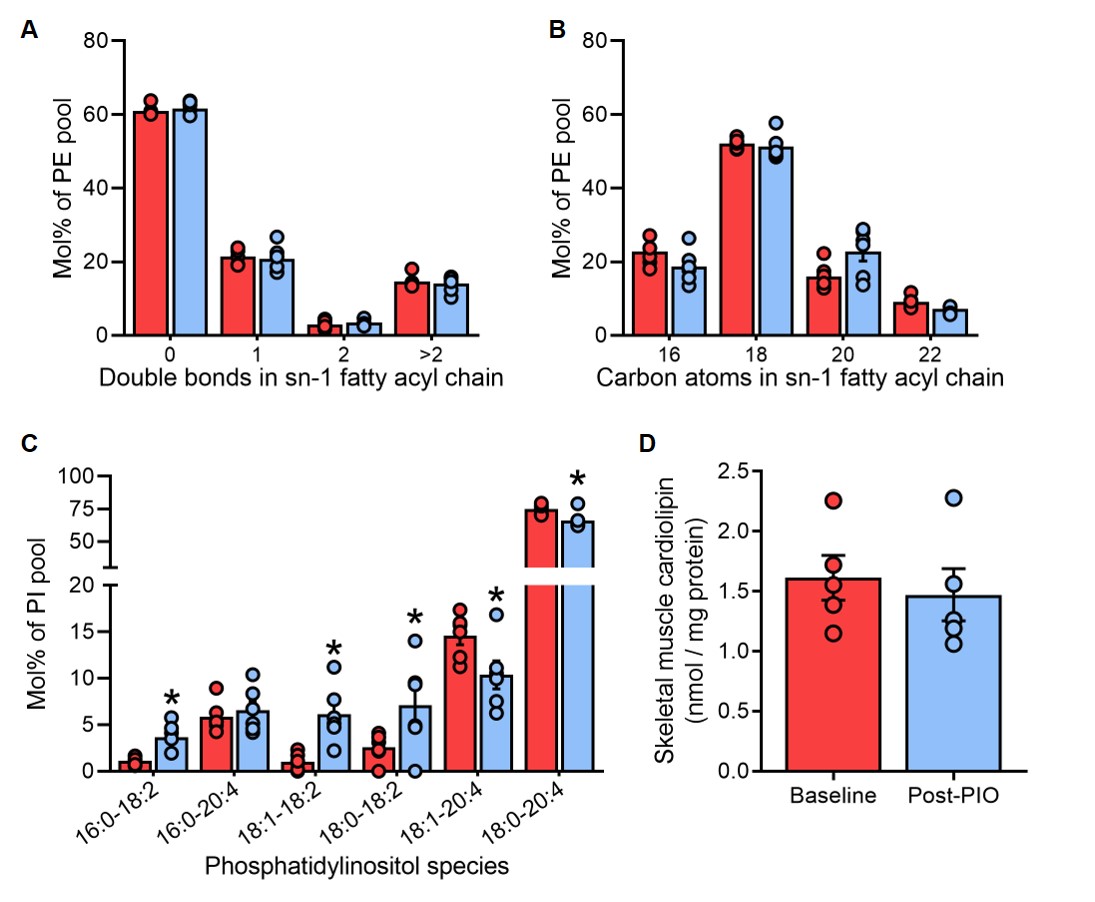

Supplement: Supplementary Figure 4 — Saturation [number of double bonds; (A)] and length (number of carbon atoms; (B) of fatty acyl chains in the sn-1 position of phosphatidylethanolamine; Fatty acyl composition of phosphatidylinositol (C) in adipose tissue, expressed as a molar fraction of total lipids in respective class. Skeletal muscle total cardiolipin content (D). Data are mean±standard error (filled bars) and individual values (filled circles) for n=6 (A–C) or n=5 (D) subjects. [file Image_4.JPEG]
